# Genetic diversity, molecular phylogeny and selection evidence of the silkworm mitochondria implicated by complete resequencing of 41 genomes

**DOI:** 10.1186/1471-2148-10-81

**Published:** 2010-03-24

**Authors:** Dong Li, Yiran Guo, Haojing Shao, Laurent C Tellier, Jun Wang, Zhonghuai Xiang, Qingyou Xia

**Affiliations:** 1 The Key Sericultural Laboratory of Agricultural Ministry, College of Biotechnology, Southwest University, Chongqing 400715, China; 2 BGI-Shenzhen, Shenzhen 518083, China; 3 Department of Biology, University of Copenhagen, Universitetsparken 15, 2100 Kbh Ø, Denmark; 4 Institute of Agronomy and Life Sciences, Chongqing University, Chongqing 400030, China

## Abstract

**Background:**

Mitochondria are a valuable resource for studying the evolutionary process and deducing phylogeny. A few mitochondria genomes have been sequenced, but a comprehensive picture of the domestication event for silkworm mitochondria remains to be established. In this study, we integrate the extant data, and perform a whole genome resequencing of Japanese wild silkworm to obtain breakthrough results in silkworm mitochondrial (mt) population, and finally use these to deduce a more comprehensive phylogeny of the Bombycidae.

**Results:**

We identified 347 single nucleotide polymorphisms (SNPs) in the mt genome, but found no past recombination event to have occurred in the silkworm progenitor. A phylogeny inferred from these whole genome SNPs resulted in a well-classified tree, confirming that the domesticated silkworm, *Bombyx mori*, most recently diverged from the Chinese wild silkworm, rather than from the Japanese wild silkworm. We showed that the population sizes of the domesticated and Chinese wild silkworms both experience neither expansion nor contraction. We also discovered that one mt gene, named *cytochrome b*, shows a strong signal of positive selection in the domesticated clade. This gene is related to energy metabolism, and may have played an important role during silkworm domestication.

**Conclusions:**

We present a comparative analysis on 41 mt genomes of *B. mori *and *B. mandarina *from China and Japan. With these, we obtain a much clearer picture of the evolution history of the silkworm. The data and analyses presented here aid our understanding of the silkworm in general, and provide a crucial insight into silkworm phylogeny.

## Background

Mitochondria have a genetic system which is different and distinct from the nuclear genome. They have a rapid tempo of nucleotide substitution and present a special form of maternal inheritance [1]. The domesticated silkworm, *B. mori*, is the model organism of Lepidoptera, which has been producing industrial silk for humans over a period of more than 5,000 years [2]. It embraces more than 1,000 inbred strains worldwide [3], and has recently been serving as a bioreactor [4]. There are two major populations of *B. mandarina*: notably the Chinese, with a haploid chromosome number of 28 (equal to those of *B. mori*), and the Japanese, with a haploid chromosome number of 27. They have different geographic distributions [3]. In 2006 Arunkumar et al. [5] analyzed six complete mt genomes to construct the phylogeny for the Bombycidae family, and although the concurrence of their methods found the Chinese *B. mandarina *to be the progenitor species of *B. mori*, some of their methods (ML phylogeny) also tentatively proposed that Chinese and Japanese *B. mandarina *share a clade, belonging to a single, monophyletic group on the phylogenic tree, distinct from *B. mori*. Based on the same data, Pan et al. [6] concurred with the consensus of Arunkumar et al., that the Japanese *B. mandarina *is a more distant taxon from the domesticated silkworm than the Chinese *B. mandarina*.

A clearer picture for the Bombycidae phylogeny in confirmation of this became desirable. Here, we take advantage of 41 mt genomes (covering both wild silkworm groups and the domesticated silkworms) to again infer the ancestor of *B. mori*, and arrive at compelling evidence confirming the conclusions of that the apparent progenitor of *B. mori *is the Chinese *B. mandarina*, in confirmation of both Arunkumar et al and Pan et al.

The domesticated silkworm has evolved to be completely dependent on humans for survival, and many domestication-related genes have been found to be selected for, when comparing the domesticated silkworm with the Chinese wild silkworm, accounting for 2.9% of the genome-wide genes [7]. This remarkable footprint by selection effect implied a potential selection imprint left in the silkworm mt genome. Moreover, previous studies have demonstrated that variants in mt genome were often subjected to selection [8-10]. With availability of genome-wide resequencing data from 40 silkworms, here we have the opportunity to make numerous comparisons and to investigate the phylogeny of silkworm mitochondria, in order to study whether the silkworm mt population has experienced any expansion or contraction, and to detect the selection effect shaping on the mt genome.

## Methods

### Whole genome resequencing of Japanese wild silkworm

The pupa of a single individual of Japanese wild silkworm was used to extract genomic DNA, based on a standard protocol for genomic DNA extraction. We followed the manufacturer's instructions (Illumina) to prepare DNA library. Briefly, we made it by following the workflow on Illumina Genome Analyzer II system: cluster generation, template hybridization, isothermal amplification, linearization, blocking, and denaturization and hybridization of the sequencing primers. Then the Illumina base-calling pipeline (SolexaPipeline-0.3) was applied to get sequences from the fluorescent images. Short read data have been deposited in the NCBI Short Read Archive under the accession number SRA009886.

### Public data availability

Through NCBI Short Read Archive http://www.ncbi.nlm.nih.gov/Traces/home/ with accession number SRA009208, we downloaded all of the 40 silkworm resequencing data (see Table 1), including 29 domesticated silkworms and 11 Chinese wild ones, detailed information of which can be found elsewhere [7], the silkworm nuclear reference genome sequence was derived from the SilkDB [11] (http://silkworm.swu.edu.cn/silkdb/ or http://silkworm.genomics.org.cn/). From GenBank http://www.ncbi.nlm.nih.gov/ we obtained three mt genome sequences (accession numbers AB070264, AY301620, and NC_003395) and their annotations, which were serving as references. We used these reference genomes in all of our consensus assembly below.

**Table 1:** The detailed information of samples and sequencing summary

Sample ID	Name of strain	System or location	Effective Depth (X)	Genome coverage (%)	DifferenceRate (%)
D1	J7532	Japan	68.98	99.62	0.48
D2	J04-010	Japan	13.04	96.33	0.56
D3	J872	Japan	36.28	99.90	1.27
D4	J106	Japan	9.14	95.88	0.51
D5	N4	Japan	77.62	99.92	1.54
D6	Cambodia	Cambodia	32.67	99.98	0.65
D7	Lao II	Laos	12.88	97.42	1.32
D8	India M3	India	51.2	99.22	0.44
D9	Europe18	Europe	23.82	99.46	0.51
D10	Italy16	Italy, Europe	24.12	99.46	0.35
D11	Soviet Union NO.1	Former SU, Europe	10.98	94.75	0.41
D12	15-010	Mutation	8.99	93.71	1.28
D13	02-210	Mutation	51.37	99.96	0.43
D14	15-001	Mutation	49.61	99.72	1.36
D15	Mutation M_2_	Mutation	22.03	99.62	1.32
D16	A06E	Guangdong province, China	75.04	99.71	1.52
D17	Damao	Sichuan province, China	28.66	99.11	0.64
D18	Ankang NO.4	Shanxi province, China	79.93	99.91	0.62
D19	ZT500	Gansu province, China	165.42	99.99	0.61
D20	Zhugui	Zhejiang province, China	77.61	99.77	0.43
D21	Bilian	Jiangsu province, China	62.83	99.49	1.4
D22	ZT900	Sichuan province, China	80.81	99.61	1.05
D23	ZT100	Hunan province, China	89.7	99.83	0.41
D24	Sihong	Jiangsu province, China	105.03	99.75	0.31
D25	Xiaoshiwan	Zhejiang province, China	20.46	98.31	0.59
D26	C108	Chongqing, China	27.57	92.66	0.4
D27	Sichuang M_3_	Sichuan province, China	51.3	99.67	0.58
D28	ZT000	Guizhou province, China	166.85	99.96	0.53
D29	Handan	Hebei province, China	51.94	99.90	0.32
W1	*B. mandarina *Ziyang	Sichuan province, China	27.6	96.07	2.49
W2	*B. mandarina *Nanchong	Sichuan province, China	106.15	98.18	2.46
W3	*B. mandarina *Hongya	Sichuan province, China	25.27	94.18	1.94
W4	*B. mandarina *Pengshan	Sichuan province, China	76.03	95.45	1.95
W5	*B. mandarina *Ankang	Shanxi province, China	30.54	92.30	2.01
W6	*B. mandarina *Yichang	Hubei province, China	108.98	96.63	2.77
W7	*B. mandarina *Yancheng	Jiangsu province, China	72.06	97.10	2.03
W8	*B. mandarina *Luzhou	Sichuan province, China	40.54	92.45	2.33
W9	*B. mandarina *Hunan	Hunan province, China	114.57	96.91	2.03
W10	*B. mandarina *Suzhou	Jiangsu province, China	49.11	95.40	2.05
W11	*B. mandarina *Rongchang	Chongqing, China	79.31	95.89	2.04
W12	*B. mandarina *Japan	Hokkaido, Japan	13.52	81.47	0.81
Total	-	-	2319.56		

AB070264 represents the strain C108, which served as the domesticated population reference. AY301620 was used as the Chinese wild mitochondria reference, which is from Ankang in Shanxi Province of China [6]. NC_003395 is a Japanese wild individual from Tsukuba, Ibaraki, Japan [12], and we map our resequencing data (Japanese wild silkworm) onto it. Mt genome sequence of *Antheraea pernyi *(NC_004622) and *Eriogyna pyretorum *(NC_012727) were also downloaded to be applied as outgroup when reconstructing phylogeny.

### Reads alignment and consensus assembly

Although we only included the mt genome sequence in the analysis, we prefer to map raw short reads onto the whole genome [including nuclear DNA sequence and mitochondrial DNA (mtDNA) sequence], in order to make alignment more reliable. Due to the fact that three sorts of resequencing data of silkworm have been in hand - which are the domesticated group, the Chinese wild population, and the Japanese wild population - there must be three different references to be used by program SOAP v1.09 [13]. So we built three different reference genomes which are the silkworm nuclear reference genome sequence with C108 mtDNA sequence, with Chinese wild mtDNA sequence and with Japanese wild mtDNA sequence. The mapping results showed that Chinese wild silkworms have a higher mismatch rate than the domesticated ones (see Table 1), which suggests a high sequence diversity in Chinese wild group, even in the intraspecific comparison between Chinese wild variety Ankang and the Chinese wild reference (which is also from Ankang in Shanxi Province of China). Followed by SOAPsnp [14], which based on Bayesian theory, we calculate the posterior probability of each possible genotype at every genome position, from the alignment results for each sample. Then the consensus was structured by the highest probability. 41 consensus sequences have been put into the Genbank under accession number GU966591-GU966631.

### SNP detection and experimental validation

We used SOAPsnp [14] to call SNPs for each variety. After setting

-u -r 0.01 -m,

six steps were used to filter out the unreliable variants: 1) we set Q20 quality cutoff; 2) two unique reads were allowed at least; 3) the SNPs had to be at least 5 bp away from each other; 4) the approximate copy number of flanking sequences had to be no more than 2; 5) *P *value of the rank sum test had to be no less than 0.05; 6) the number of unique mapped reads had to be greater than or equal to half of the number of total mapped reads.

To evaluate our SNP calling strategy, we randomly selected 50 sites for PCR-Sanger dideoxy sequencing validation using the AB 3730XL. After manually checking all the intensity trace files, we found that all the sites were confirmed by the PCR-sequencing results.

### Linkage Disequilibrium (LD) measure calculation

To measure LD level in the silkworm mt population, we used the normalized measure of allelic association estimate *D' *[15], which can not be easily influenced by rare alleles examined [16]. We set the parameters in the software Haploview [17] as follows:

-maxdistance 200-dprime-minGeno 0.6-minMAF 0.1-hwcutoff 0.001. Then spot chart was plotted with R scripts which drew averaged *D' *against pairwise marker distance.

### Phylogeny reconstruction

Five silkworm mt sequences (with accession number AB070264, AY301620, NC_003395, NC_004622, and NC_012727) were firstly aligned using MUSCLE v3.7 [18] with default settings, adjusting coordinates for the three resequencing groups. From this, we got 46 mt genomes, after integrating these five sequences and 41 consensus that we have, to perform phylogenetic reconstruction following MEGA v4 [19] by using Neighbour-Joining method and Mrbayes v3.1.2 [20] under the Bayesian theory. In Mrbayes, we chose the GTR+gamma+I model, and set the chain length to 50,000,000 (1 sample/1000 generations) and burned in the first 10,000 samples. Almost identical results were obtained in two independent runs. The quality of being dependable of the NJ trees was bootstrapped with 1000 replicate estimates. To evaluate the confidence level in the tree selection, we applied statistical tests with CONSEL [21]. The site-wise likelihood file is derived from PhyML calculatation [22].

### Tests of effective population size

First, using the SNP information of each group, we inferred the mt sequence mismatch distributions for domesticated group and Chinese wild group, where a ragged distribution implies the stable population size, and where a bell-shaped pattern is often related to population growth [23,24]. Further, Tajima's *D *and Fu and Li's *D *tests for population size were also performed with DnaSP v5 [25].

### Neutrality tests

It became clear to us that, during the domestication process of the silkworm, 354 genes in the nuclear genome bear a strong human selection footprint [7]. The mt genome may therefore also experience selective pressure. In order to examine mtDNA protein evolution, we compare the rate of nonsynonymous to synonymous mutation within and between species in each gene. If these ratios differ significantly, they provide evidence of selection pressure [26]. We then applied Williams' correction to calculate the G statistic [27]. Moreover, we also preformed the two likelihood ratio tests (LRTs), based on widely used branch-site models from PAML 4.2 [28], to detect any positive selection.

## Results

### Determining mapping strategy and mapping summary

Before applying the strategy of mapping raw reads onto different references, first round mapping was initiated, which only took a single mt genome C108 as a reference to be mapped. After SNP calling, there was an obvious separation among these three groups in phylogeny analysis (data not shown). This impressed us that we should use different reference datasets based on silkworm classification as a reference for each group, so that we can get more realistic genetic variation. Before we clearly acknowledge the separation among these three populations, it is essential to perform these alignments. Here, we just take the mt sequence into consideration; however, we must introduce the nuclear DNA sequence, because there are some similar regions between the mt sequence and nuclear genome. A sequencing read would have more than one hit mapped to the whole genome (the mt genome plus the nuclear genome), while it would be with unique hits to the mt sequence when we excluded the nuclear DNA sequence. The latter, exclusive mt genome situation would interrupt meaningful SNP detection [14].

After aligning short reads onto the reference genome by using the SOAP alignment program [13], we obtained a 2319.56 fold effective depth for all the 41 varieties and each ranged from 8.99 to 165.42 fold. The minimal depth 8.99 fold covering 93.71% of the mt genome (see Table 1) suggests that there was sufficient content to detect the variation. Though this fluctuation in depth was apparent, we found there was no significant difference [Mann Whitney U (MWU), *P *= 0.36] when one takes the depth between domesticated silkworms and wild ones into comparison.

### Mitochondria diversity and the higher polymorphism level in wild silkworm

We then used SOAPsnp package [14], which checks the read quality and mapping positions to detect SNPs. Filtered by the criteria described in Method Section, we identified 88 SNPs when taking 29 domesticated mt sequences as a whole, 231 SNPs in Chinese wild population, and 46 SNPs in Japanese wild group (Table 2). We totally identified 347 SNPs for our 41 samples in brief. The randomly chosen subset of the SNPs was sequenced with PCR, and we found they are all consistent with the genotype calling. The polymorphism level (θ_π_) of mt sequence among Chinese wild population (6.20 × 10^-3 ^nucleotide differences per site) is more than six times that among domesticated varieties (1.14 × 10^-3^) (Table 2), the relative larger reduction in polymorphism is most likely caused by inbreeding or population bottlenecking. Similar phenomenon was revealed in the nuclear DNA analyses [7]. However, the Japanese wild population, which only distributes in Japan and Korea [3], has a moderate sequence diversity (2.02 × 10^-3^), suggesting that it has a small effective population size compared to Chinese wild silkworm. This conclusion must be tempered somewhat by the low Japanese sample number.

**Table 2:** Statistical summary for three different groups.

Data set	Effective Length (bp)	N	S	MPSD	θ_π_
Domesticated silkworms	12535	30	88	14.26	1.14 × 10^-3^
Chinese wild silkworms	13461	12	231	83.50	6.20 × 10^-3^
Japanese wild silkworms	11369	2	46	23.00	2.02 × 10^-3^

Numbers of Sequence (N), Segregating Sites (S), Mean Pairwise Sequence Differences (MPSD) and Nucleotide Diversity (θ_π_).

### No recombination event in silkworm mt genome

**Figure 1:**
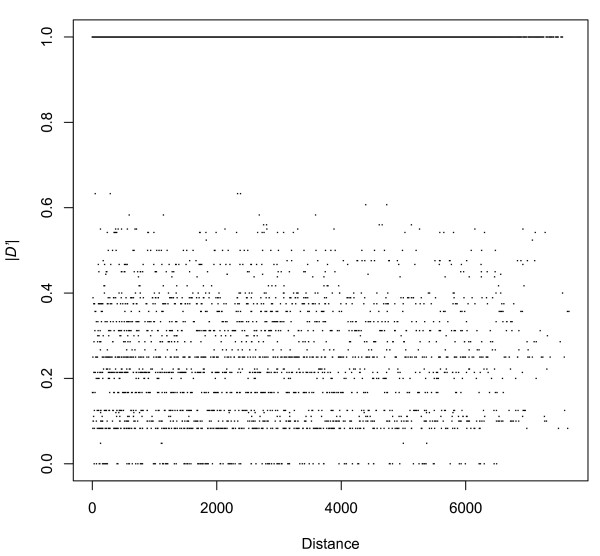
**The relationship between linkage disequilibrium, measured by |*D'*|, versus distance between sites for all the 11 complete Chinese wild mitochondrial genomes**.

It is a point of frequent contention whether the mt genome undergoes recombination or not [29]. We used *D'*, shown to be less sensitive to the effects of the allele frequency variation [30] than others measures, to assess the linkage disequilibrium (LD) rates in the domesticated silkworm and Chinese wild silkworm varieties, and to check whether silkworm mt genome has experienced recombination. No marker in the domesticated group passed all the thresholds set by Haploview, but 146 out of 231 markers have passed all criteria in the Chinese wild group, which could be applied to analyze the allelic association. Of the 10,585 pairs of the 146 polymorphic sites, 8,301 showed the maximum LD rate (|*D'*| = 1) (Figure 1). High proportion (78.4%) result suggested no evidence for the recombination in the silkworm mt genome.

Furthermore, there was also no correlation between genome distance and |*D'*|, and distribution of site pairs with |*D'*| < 1 were randomly distributed with physical distance (Pearson's correlation coefficient, ρ = 5.31 × 10^-3^; *P *= 0.80). Although this is the first investigation for silkworm mitochondria recombination, there seems to be only limited occurrence, making it unnecessary to consider its effect on population structure or evolution.

### Phylogenetic analysis

**Figure 2:**
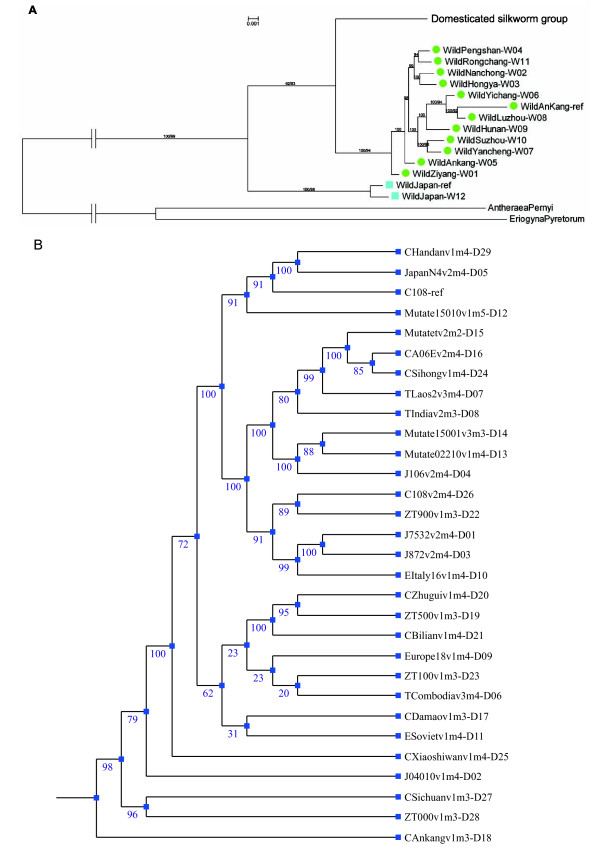
**Silkworm mitochondrial phylogeny**. (A) Phylogeny in different silkworm groups, based on whole mitochondrial genome. The tree was built using Bayesian method with GTR+gamma+I model. Bootstrap values measured by the posterior probabilities are shown at the nodes. We also reconstructed a Neighbour-joining tree in Mega, using the same consensus sequence. The support values (with 1000 bootstrap replicates) are listed following the posterior probabilities. Except for the outgroup *Antheraea pernyi *and *Eriogyna pyretorum*, as well as the domesticated group, each sample is represented by a combination of symbols representing respective silkworm groups (filled circles for Chinese wild group and boxes for Japanese wild group), sample names and sample IDs ("W01" to "W12", "WildAnKang-ref" for the reference genome of Chinese wild group, "WildJapan-ref " for the reference genome of Japanese wild group). The detailed information for samples could be found in Table 1. (B) More fine scale phylogeny for the domesticated silkworm using Mrbayes. For the tree has a very short branch length, only topology was shown in this picture. The posterior probabilities are listed at each node. Each sample is represented by sample names and sample IDs ("D01" to "D29", "C108-ref" for the reference genome of domesticated group).

To quantify population structure, we used all the SNPs identified, and all the specific sites among groups, to reconstruct an accurate illustration of population history, which could distinguish the ancestor from which the domesticated silkworm was most recently bifurcated. Both Bayesian and Neighbour-joining methods were used to confirm the topology accuracy of the estimated phylogenetic tree, and similar results were obtained using different approaches. So, we only show here the Bayesian tree in Figure 2A and Figure 2B. Although some efforts have been made to elucidate the phylogeny of silkworms based on mtDNA sequence [5,6], here, it is also essential to perform a genome-wide analysis which includes more individual sequences, and which would get more comprehensive picture of silkworm clade. Using the mt genome sequences of *Antheraea pernyi *and *Eriogyna pyretorum *as the outgroup, we can see clearly and conclusively that the domesticated silkworm is closer to the Chinese wild silkworm than to the Japanese wild silkworm. Also, the high bootstrap values in topology provide compelling evidence that domesticated silkworm mtDNA was more recently bifurcated from the Chinese wild group, rather than from the Japanese wild group.

To evaluate the level of confidence in tree selection, we test whether alternative placements of Japanese wild silkworm among the Bombycidae could be accepted on basis of the approximately unbiased (AU) test and the weighted Shimodaira-Hasegawa (WSH) test [31]. With our dataset, they reject all the alternative scenarios (*P *< 1 × 10^-6^). As mentioned in the Method section, the reference of the domesticated and Chinese wild group is from C108 and Ankang, respectively, but another individual from these two lines did not cluster together with its references (Figure 2A and Figure 2B). This result suggests the intraspecific mtDNA sequence diversity would be higher than the interspecific diversity.

### Stable population size in domesticated and Chinese wild population

**Figure 3:**
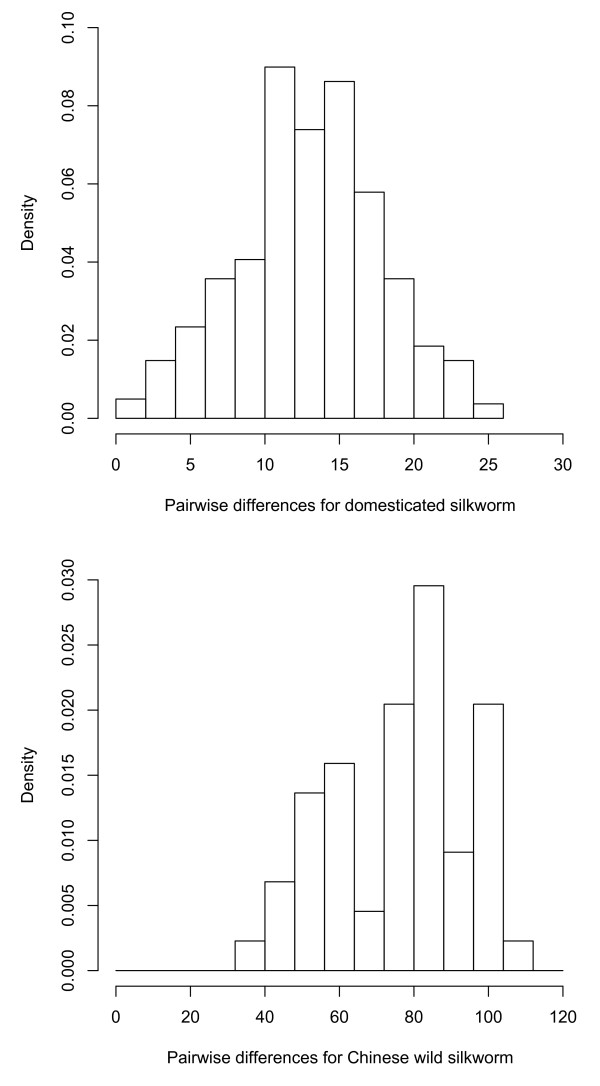
**Pairwise difference distribution for domesticated silkworm genome sequences (top) and Chinese wild silkworm genome sequences (bottom)**.

Previous studies have deduced that the domesticated silkworm effective population maintained a stable size posterior to the domestication event [7]. The frequency distribution of pairwise differences for domesticated and Chinese wild group all showed ragged pattern (Figure 3), indicating their effective population sizes remain constant [23,32]. Based on the number of segregating sites and the nucleotide diversity, we further performed Tajima's *D *[33] and Fu and Li's *D *[34] tests. They both show no indication of population expansion or contraction in the two groups (Tajima's *D*_wild _= -0.41308, *P *> 0.10; Fu and Li's *D*_wild _= -0.33548, *P *> 0.10; Tajima's *D*_domesticated _= -1.65367, *P *> 0.05; Fu and Li's *D*_domesticated _= -2.19707, *P *> 0.05).

### Positive selection detection

There is no recombination event in the Chinese wild silkworm mitochondria, and the population size of domesticated silkworm and Chinese wild silkworms both remain stable. So it is reasonable to infer selection forces shaping mtDNA by using McDonald-Kreitman test for each gene. The result showed the *cytochrome b *(*cytb*) gene had a significant (*P *= 1.8 × 10^-3^) deviation from neutrality (Table 3), indicating it has been effected by positive selection posterior to silkworm domestication [26].

**Table 3:** Number of replacement and synonymous substitutions within and between *B. mori *and *B. mandarina*

	Fixed between species	Polymorphic within species	Total
Synonymous	10	16	26
Replacement	21	5	26
	31	21	G_adj_* = 9.75
			P = 1.8 × 10^-3^

* Williams correction was applied to calculate the G statistic (Sokal and Rohlf 1994)

We also looked for positive selection using branch-site likelihood ratio tests for this gene, showing significant difference (*P *= 0.014) between the foreground and background. These two kinds of independent tests both show statistical power in this scenario, so we can believe the *cytb *gene is important to the domesticated silkworm, which will be elucidated in the discussion.

## Discussion

The silkworm is the best-characterized model for biochemical, genetic, and genomic studies of the order Lepidoptera, and for insect domestication. Here we report a full-scale genome-wide mitochondrial map, which includes all the major silkworm classes in Bombycidae, and unveils - at a single base-pair resolution - the distinction between the two wild silkworm subgroups. This map will provide a valuable resource for further study on silkworm mitochondria evolution, and will accelerate the research of functional identification among silkworm genes.

We estimated θ_π _values for SNPs in mitochondria and found they were 1.14 × 10^-3 ^and 6.20 × 10^-3 ^in *B. mori *and *B. mandarina*, respectively, and we also know that θ_π _values in the nuclear genome were 1.36 × 10^-2 ^and 1.53 × 10^-2 ^in *B. mori *and *B. mandarina*, respectively [7]. The relatively larger polymorphism level increase in nuclear genome is mainly due to the background transposable element content [35] and rapid decay of LD [7]. The pattern in humans, whose mutation rate is 2.8 × 10^-3 ^[36] and 0.8 × 10^-3 ^[37] in our mt and nuclear genomes, respectively, provides an interesting contrast to that of the silkworm. But compared to *Drosophila simulans*, we found a similar phenomenon of a nuclear genome mutation rate (1.8 × 10^-2^) [38] being higher than the mitochondrial rate (1.1 × 10^-3^) [8]. This indicated that the insect nuclear genome could tolerate more alteration, and the differential of effective population size between nuclear genome and mt genome would be more distinct in the insect. Although the diversity of mitochondria was smaller than that of the nuclear, the difference between the domesticated silkworms and the wild silkworms was apparent, which suggested they had exclusive genetic background, and local inbreeding had produced more effect on the domesticated population.

In recent years, scientists have only used limited samples or partial genome datasets to infer the phylogenic relationship amongst silkworm clades. The main features of these analyses have been on the basis of incomplete genetic information. Although it is possible for them to reach the conclusion that the true ancestor of the domesticated silkworm is the Chinese wild group, representatives of several basal lineages remain missing. Here, taking advantage of 46 complete mt genomes, we can confidently verify the consensus phylogeny of Arunkumar et al and Pan et al that *B. mori *was domesticated from the Chinese *B. mandarina*. Both the AU test and WSH test also provided further support on this argument. We also endeavored to study the major genetic characteristics left by silkworm domestication process, such as the different level of polymorphisms within each group, and the selection force shaping on the mt genome. In the mitochondrion of eukaryotes and in aerobic prokaryotes, the *cytb *protein is a component of respiratory chain complex III, and is related to electron transfer. Mutation in the site 318 of human *cytb *gene causes severe deficiency in the respiratory chain enzyme involving in patient exercise intolerance [39]. In silkworms, previous studies also showed that nine midgut-enriched genes related to energy metabolism under selection play a critical role in food digestion and nutrient absorption [7]. The identification of *cytb *gene indicates that mt genes probably contribute equally to energy metabolism process, and is important to the domesticated silkworm. This is the first time we sort out a gene under positive selection in silkworm mtDNA, and it may provide a new insight into the silkworm mtDNA evolution process.

The constant population size inference for *B. mori *and *B. mandarina *in China indicated that the Chinese wild silkworm led diverse and distinct lifestyles in the wild mulberry, and the domesticated silkworm retained and conserved their abundant interspecific polymorphism. These results would pave the way for breeding strategies later.

*B. mori *is not only well adapted to human handling, but is wholly dependent on humans for survival, in addition to being well differentiated trait-wise from its wild cousin. This makes silkworm domestication a distinctive event in agricultural history, deserving the same attention as domestication of livestock and crop plants; especially as it took place in a different geographical region (Asia vs. the Fertile Crescent) [40], and in a distinctly different culture from the earliest known, and better studied domestication events.

## Conclusions

Based on whole genome comparative analysis, with 41 silkworm mitochondrion and 5 other available mt genome sequences, we identified 347 SNPs in the silkworm mt genome. Using this data, we infer that the silkworm mt genome did not undergo recombination, and find it very strongly implied that the Chinese *B. mandarina *is the most recent ancestor of domesticated *B. mori*, in confirmation of the conclusions of Arunkumar et al and Pan et al. It is found that, after silkworm domestication and group divergence, the effective population sizes in both groups remained constant, and that identifiable genes underwent powerful selection pressures in *B. mori*.

These comprehensive overviews will provide new insight into the evolution of the silkworm. It is believed that the silkworm domestication event is of comparative significance equal to the concurrent domestication events of crop plants and animal livestock, during the same time period of human history.

## Authors' contributions

DL designed the study, performed the data collection, carried out the molecular genetic studies, drafted and revised the manuscript. YRG and LCT revised the manuscript, and HJS participated in the data analysis. QYX and JW took part in conceiving the study and helped to revise the manuscript. ZHX coordinated the study. All authors read and approved the final manuscript.
